# The International Congress

**Published:** 1868-01-01

**Authors:** 


The International Congress.—We omitted to mention in the last No. of the Journal, that we are indebted for the original translation and abstract of papers and discussions before the International Medical Congress, to the busy pen of our distinguished colleague, Prof. J. W. Freer, who was himself present as a member of the body.

The series, as a whole, will be read with interest by those desirous of knowing whereof the medical world is thinking. The topics discussed were, in the main, of the most important character.

